# Herniated Disc Causing Foraminal Stenosis and Compression of the Unilateral Exiting Nerve Root at C5-C6: A Case Report

**DOI:** 10.7759/cureus.68163

**Published:** 2024-08-29

**Authors:** Ashwinkumar Khandge, Ankit Barosani, SomiReddy Medapati

**Affiliations:** 1 Orthopaedics, Dr. D. Y. Patil Medical College, Hospital and Research Centre, Dr. D. Y. Patil Vidyapeeth (Deemed to be University), Pune, IND

**Keywords:** laminectomy, unilateral biportal endoscopy, minimally invasive spine surgery, foraminotomy, discectomy, cervical radiculopathy

## Abstract

This case report describes the use of unilateral biportal endoscopy (UBE) for treating a 40-year-old female patient with cervical radiculopathy caused by a herniated disc at C5-C6, which had led to foraminal stenosis and nerve root compression. The patient presented with a one-year history of neck pain radiating to the right upper limb, accompanied by tingling sensations. Imaging revealed loss of cervical lordosis, disc dehydration, and a right-sided foraminal disc protrusion. The patient underwent a right-sided cervical UBE with C5-C6 discectomy and foraminotomy. Postoperatively, the pain was significantly reduced, with improvement in MacNab’s grade and visual analog scale scores for neck pain and radiating pain of the upper limb at one- and three-month follow-ups. The procedure demonstrated the effectiveness of UBE in achieving good clinical outcomes with minimal complications, such as reduced soft tissue damage, minimal blood loss, and preserved spinal stability.

## Introduction

Cervical radiculopathy, thoracic radiculopathy, and lumbar radiculopathy are the most common reasons a patient needs spinal surgery [1]. In cervical radiculopathy, the predominant causes are degenerative foramen narrowing and disc herniation [1].

Historically, open discectomy, either with or without spinal fusion, has been the standard approach for treating disc herniations. Anterior cervical discectomy and fusion (ACDF) is still widely considered the gold standard for surgical management of cervical radiculopathy [2-6]. However, alternative treatments for cervical stenosis beyond ACDF include posterior techniques such as microscopic, endoscopic-assisted, or fully endoscopic posterior decompression and discectomy procedures [7].

More recently, a newer spinal endoscopy with two portals, one for viewing, the viewing portal, and the other for instrumentation called the working portal, has been introduced. Unilateral biportal endoscopy (UBE) is an emerging technique in spinal surgeries. This spinal endoscopy underwent generational changes from transforaminal and interlaminar to stenoscopy [8]. UBE was introduced for lumbar degenerative lesions, but over time, it was used in other spinal regions like cervical and thoracic degenerative conditions [9]. Cervical UBE is a recent advancement in treating degenerative conditions. Literature was found on lumbar UBE, but few are available on cervical UBE. This case report describes our cervical UBE technique for cervical disc prolapse with foraminal stenosis and its clinical outcome.

## Case presentation

A 40-year-old female patient came to the outpatient department at Dr. D. Y. Patil Medical College, Hospital and Research Centre, Dr. D. Y. Patil Vidyapeeth, Pune, with a complaint of neck pain for one year that radiated to the right upper limb. Pain in the upper limb was associated with a tingling sensation over the lateral aspect of the shoulder, thumb, and index finger for the patient for four months. The patient gave a history of heavy weightlifting as a part of her occupation, and there was no history of any trauma or fall. There were no known comorbidities. After taking the detailed history, we examined the patient. She had paraspinal tenderness in the cervical region, the power of both upper limbs at all joints was 5 on 5, and the sensation over the right upper limb was reduced at C6 and C7 dermatomes compared to the left upper limb. The patient had been managed conservatively for three months in other hospitals with medication and physical therapy with no symptomatic relief.

**Figure 1:**
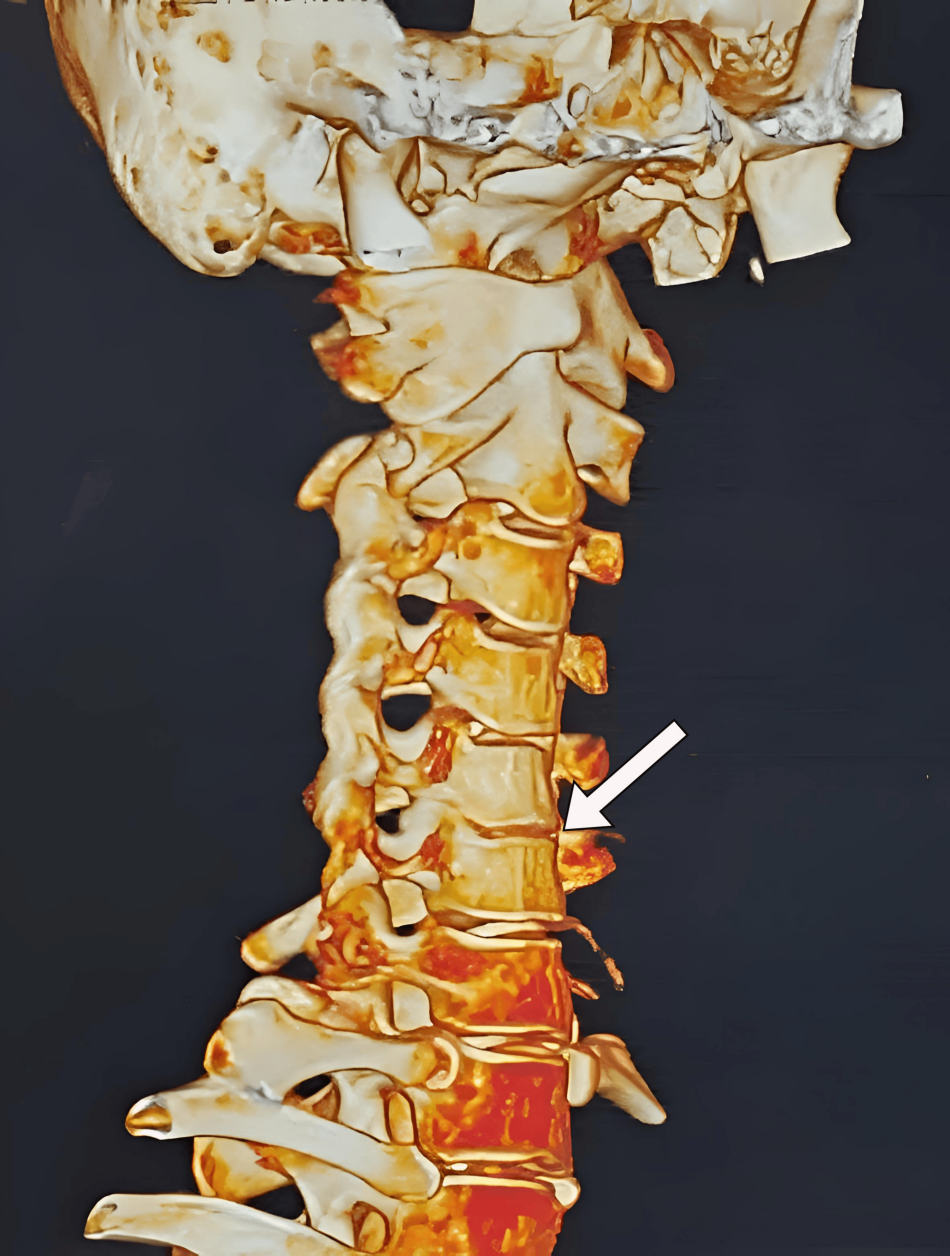
3D CT of the cervical spine. The three-dimensional computed tomography (CT) of the cervical spine showing loss of cervical lordosis and decreased intervertebral space (white arrow) between the C5-C6 level.

**Figure 2:**
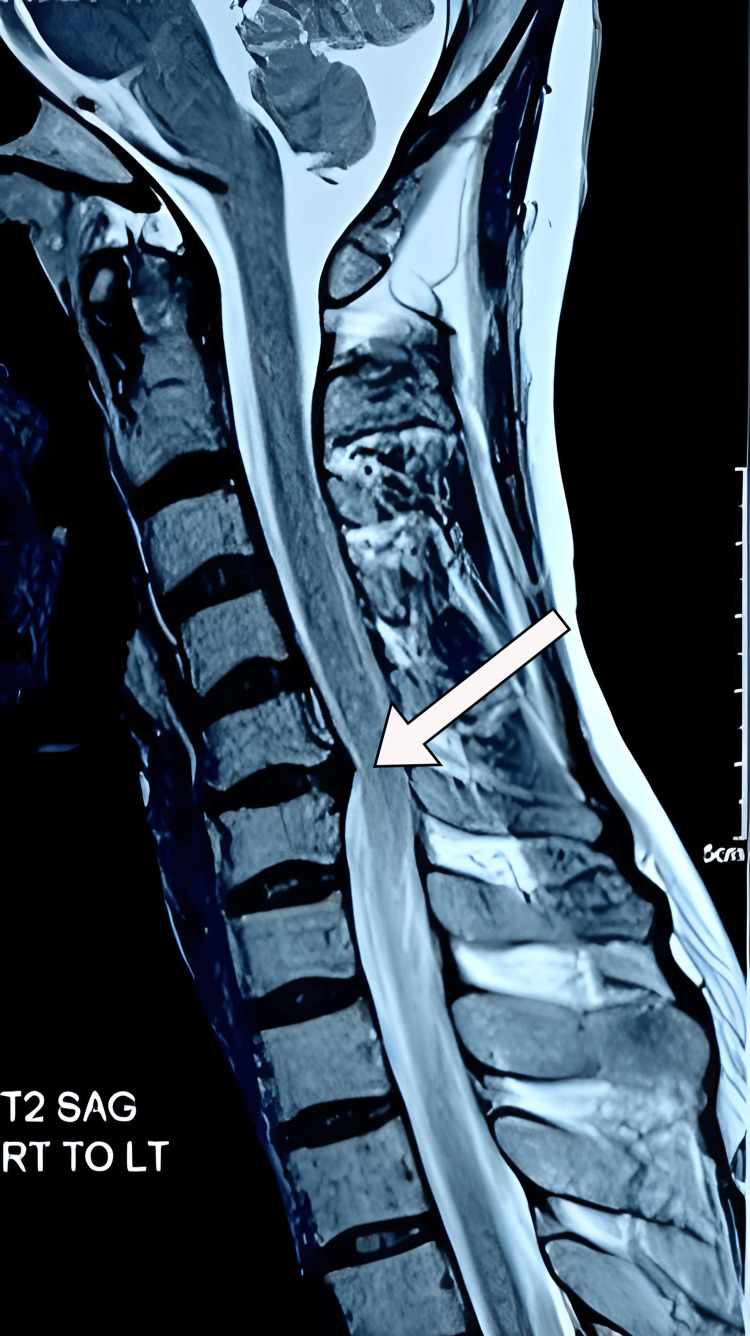
The sagittal section of T2WI of the cervical spine. The sagittal section of the cervical spine's T2 weighted image (T2WI) showing low hydration of cervical intervertebral discs and a prolapsed intervertebral disc (white arrow).

**Figure 3:**
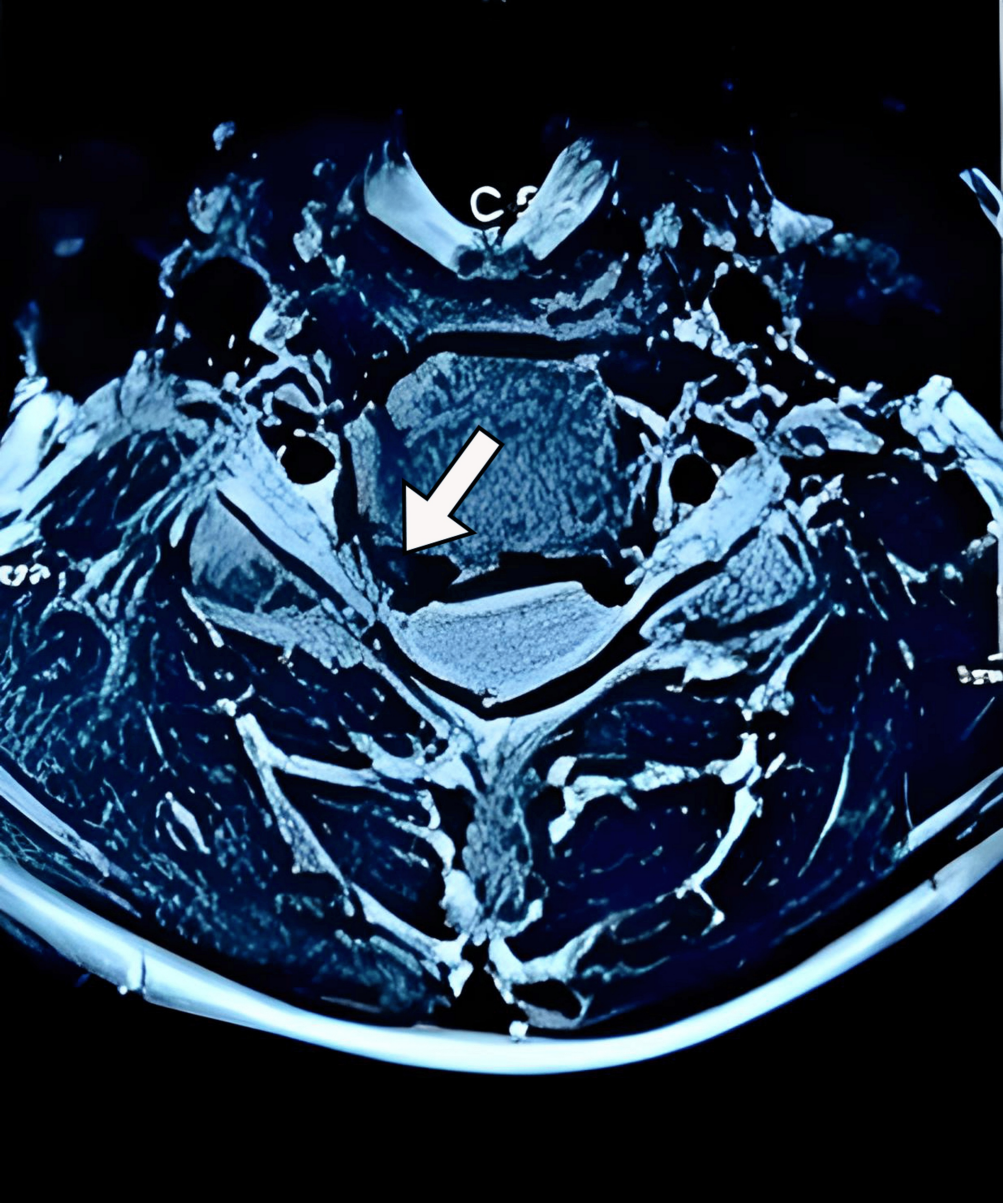
The axial section of T2WI of the cervical spine. The axial section of the T2 weighted image (T2WI) of the cervical spine showing a broad base right foraminal protrusion of the C5-C6 disc, causing right foraminal stenosis and compression of the right exiting nerve root (white arrow).

We performed an X-ray and computed tomography (CT), which showed decreased intervertebral disc space between C5-C6 and loss of cervical lordosis (Figure 1). Magnetic resonance imaging revealed low hydration of cervical intervertebral discs (Figure 2) and broad base right foraminal protrusion of the C5-C6 discs, causing right foraminal stenosis and compression of the right exiting nerve root (Figure 3). Because foraminal stenosis with unilateral radiculopathy is an indication for biportal endoscopy, we admitted the patient. We operated on her with C5-C6 discectomy and laminoforaminotomy using a UBE procedure [10].

UBE procedure

Because the patient had right-sided symptoms in this case, we surgically approached from the right side. Endoscopic instruments, including a burr, Kerrison rongeur, and various conventional spine instruments, were used, along with continuous saline irrigation and a radiofrequency (RF) probe.

Surgical setup and positioning

**Figure 4:**
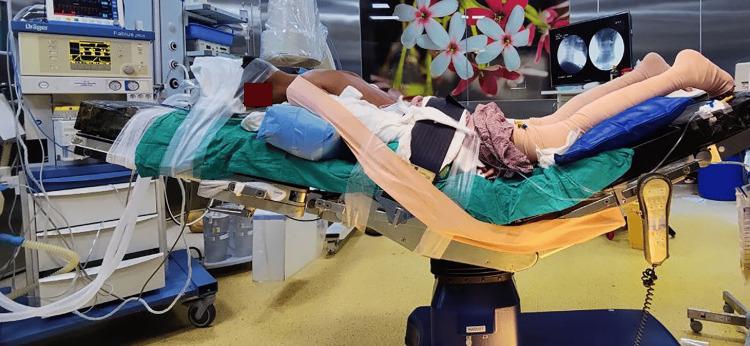
Picture showing the positioning of the cervical UBE procedure. The patient was prone and adjusted to a slight reverse Trendelenburg position with a slightly flexed head, shoulders, and arms secured with an adhesive bandage. UBE: Unilateral biportal endoscopy

The patient was positioned prone on the operation table and adjusted to a slight reverse Trendelenburg position after successful general anesthesia. We used a horseshoe headrest to stabilize the head, and the neck was slightly flexed. The head was secured with an elastic adhesive bandage. The arms were positioned caudally, and the shoulders were also pulled caudally and secured for better exposure using an adhesive bandage (Figure 4). Standard scrubbing, painting, and draping procedures were followed to prepare for surgery.

Surgical steps

Identification and Marking

**Figure 5:**
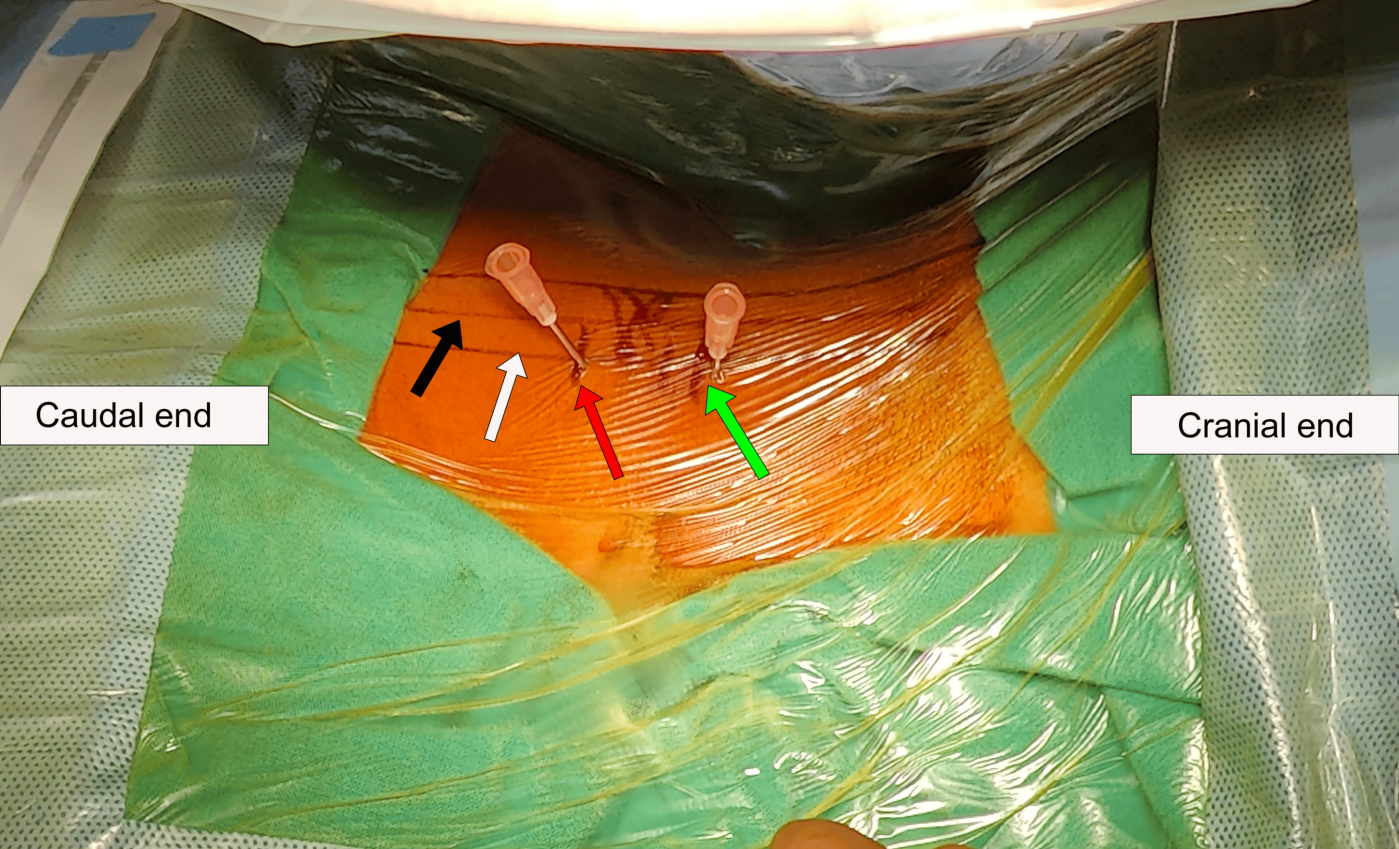
Picture with the surgical site marking. A picture with the surgical site marking showing mid spinous line (black arrow), lateral vertical line (white arrow), incision site for viewing portal (red arrow), and incision site for working portal (green arrow).

By using fluoroscopic imaging, the C5 and C6 vertebrae were identified. For surface marking, we marked a midline along the spinous process and a horizontal line at the C5-C6 intervertebral space. This horizontal line represents the level where the foraminotomy was performed. Measured approximately 2 cm lateral to the midline, a second vertical line was drawn parallel to the midline and slightly medial to the C5-C6 facet joint (Figure 5).

Because the surgeon was right-handed, the first incision was marked on the lateral line slightly cranial to its intersection to the horizontal line, which acted as a working/instrumentation portal that eased the surgical maneuvers with the right hand, allowing optimal access to the foramen and the disc space. We marked a second portal site 2 cm caudad to the working portal on the lateral line, which acted as a viewing/camera portal to optimize the approach and visualization (Figure 5).

Portal formation and instrumentation

**Figure 6:**
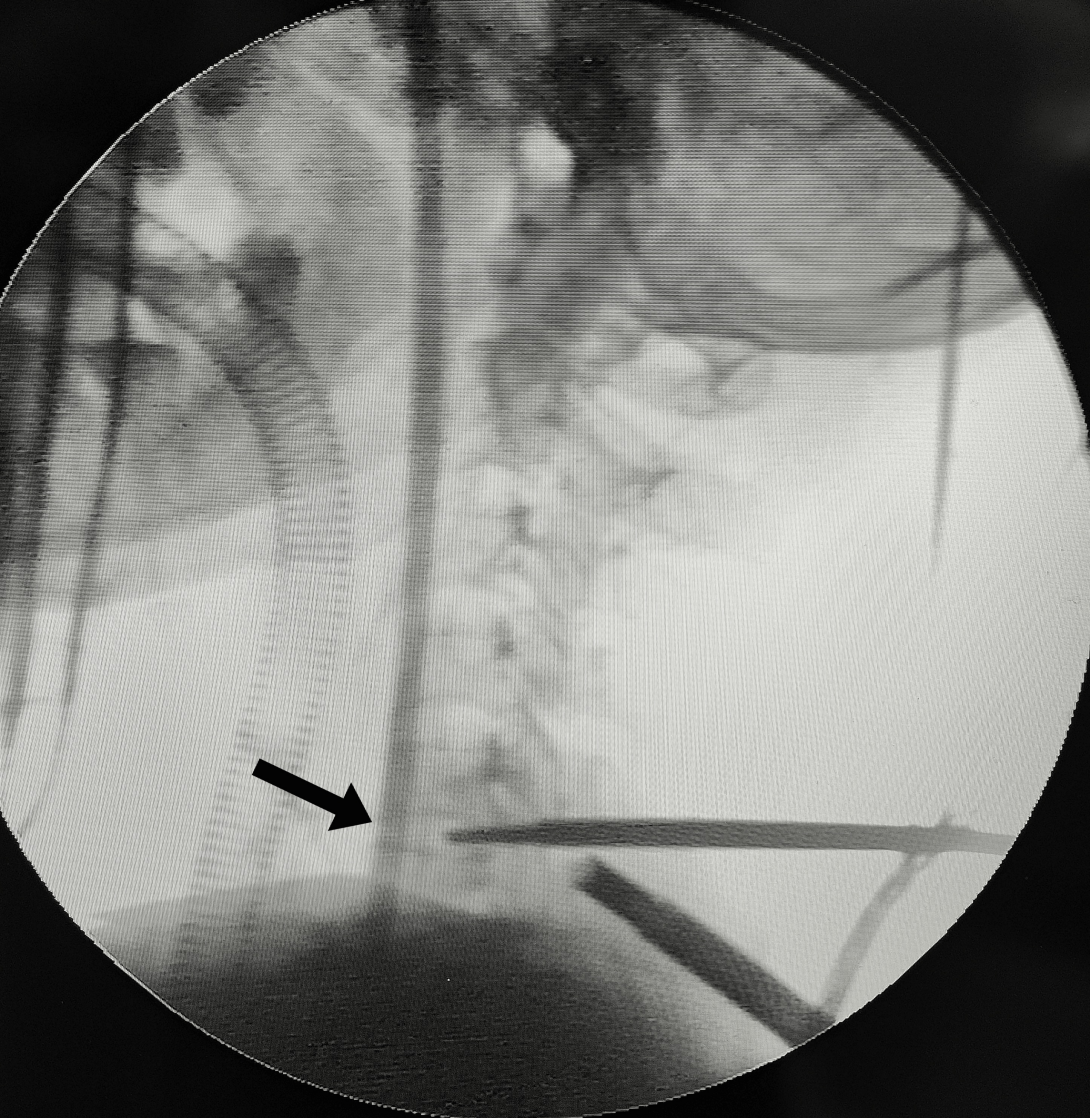
Intraoperative fluoroscopy image at the C5-C6 level. An intraoperative fluoroscopy image of the cervical spine showing the scope and a probe is used to confirm the C5-C6 level (black arrow).

**Figure 7:**
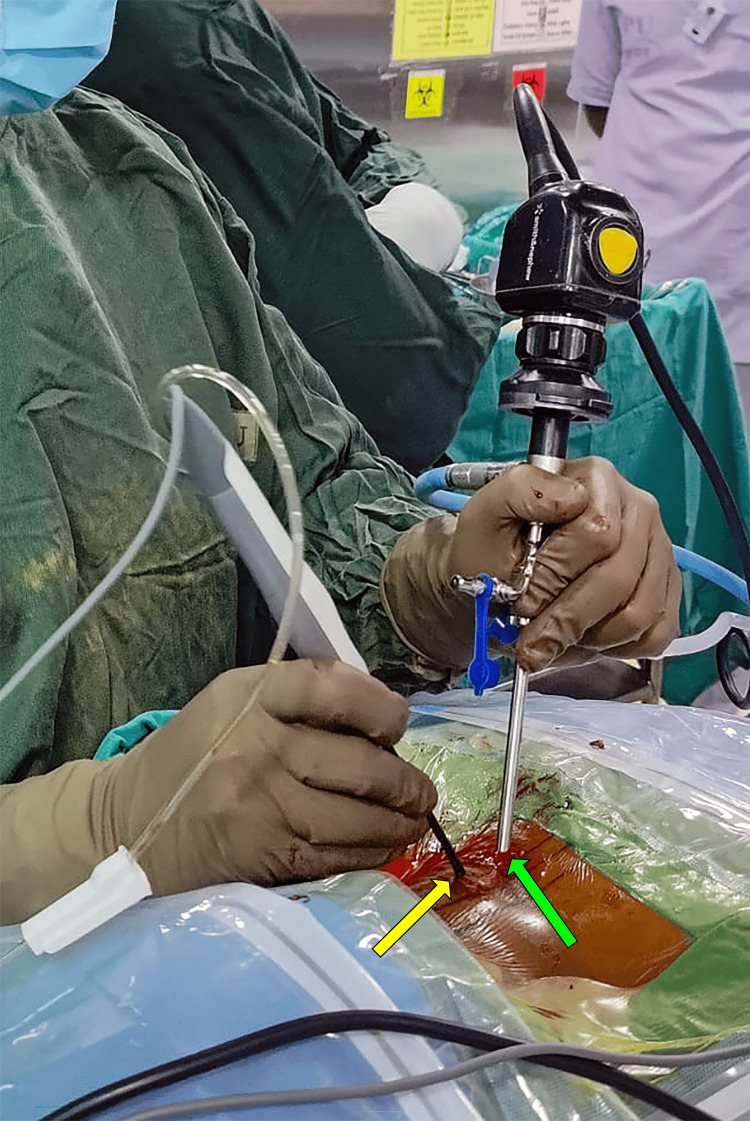
Picture of the UBE surgical setup. Picture showing the unilateral biportal endoscopy (UBE) surgical setup of viewing portal (green arrow) with 30-degree scope with a camera, a light source, and an arthroscopic saline irrigation set and a working portal (yellow arrow) with a radiofrequency (RF) wand.

Triangulation was done by C-arm fluoroscopic guidance, with 18-G needles marking the trajectory to ensure portal alignment. A 9-mm skin incision was made at the marked working portal, followed by placing an obturator to identify the inferior part of the C5 lamina and the superior part of the C6 lamina. The obturator was removed, and serial dilatations were done to 13 mm to ease the use of instruments. An endoscope was introduced through the viewing portal after the incision, saline irrigation was started, and fluoroscopic imaging was used to confirm the triangulation (Figures 6, 7).

Decompression

Various instruments were employed during the procedure, including the RF wand, a high-speed 4-mm cylindrical burr, a diamond burr, and conventional spinal instruments such as pituitary forceps, ball-point nerve hook, and Kerrison rongeurs. The RF wand was used to ablate the soft tissues, which provided access to the “V” point-the junction of the laminae of C5-C6 and the medial facet joint. This critical area comprises the lower part of the cranial laminae, the upper part of the caudal laminae, and the medial section of the facet joint. Afterward, Kerrison rongeurs and burrs were utilized to burr and remove the cranial part of the C6 lamina and the medial side of the facet joint using a high-speed 4-mm diamond burr to reveal the ligamentum flavum (LF).

LF removal

We released the LF from its attachment using a curette and double-end probe. The LF was then removed in one piece, exposing the underlying fat and dural sac and allowing clear visualization of the nerve root.

Disc removal and final steps

**Figure 8:**
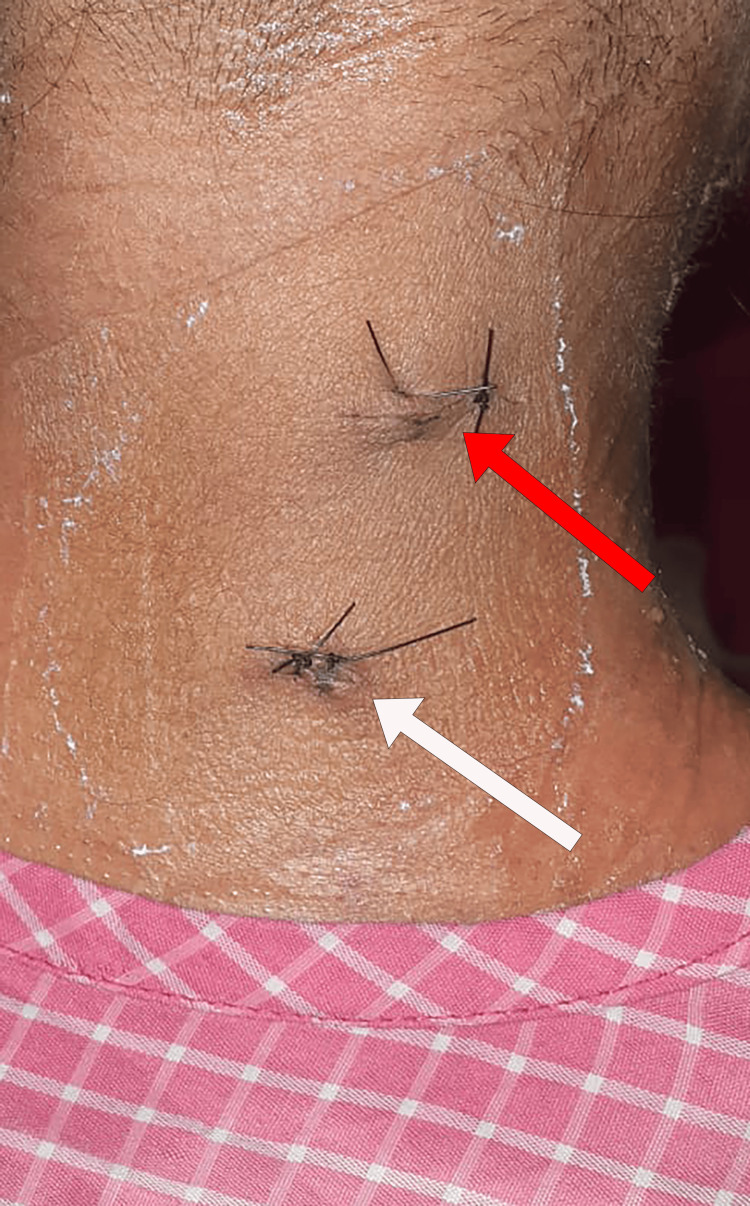
Postoperative incision site of the patient. Postoperative surgical site showing the sutured viewing (white arrow) and working (red arrow) portals.

Meticulous hemostasis was achieved using the RF wand. The C5-C6 disc protrusion was identified, and the root was retracted using a nerve root retractor. Discectomy was performed using disc forceps to decompress the exiting nerve root. All the steps were seen in the endoscopy video (Video 1). The incisions were then closed using the standard procedure (Figure 8).

**Video 1:** Video of cervical UBE. Video showing the steps of the cervical unilateral biportal endoscopy (UBE) procedure.

Postoperative course

The entire surgical procedure lasted 120 minutes with minimal blood loss and minimal tissue damage. By postoperative day 1, the patient was made to sit at the bedside and walk without assistance, and she reported only slight pain at the surgical site. The recovery process was rapid and smooth, with no significant complications such as dural tears during the procedure, incomplete decompression, transient nerve palsy, or symptomatic postoperative spinal epidural hematoma.

At their one- and three-month follow-ups, the patient was evaluated according to the visual analog scale (VAS) of radiating pain to the upper limb and neck pain and MacNab’s grade. Postoperative values at one month and three months were compared to the preoperative period. VAS score for neck pain improved from 6 pre-op to 2 at the one-month follow-up and 1 at the three-month follow-up. VAS score for radiating pain improved from 7 pre-op to 0 at the one-month and three-month follow-ups. MacNab’s grade also improved from fair pre-op to excellent post-op.

## Discussion

Cervical radiculopathy, thoracic radiculopathy, and lumbar radiculopathy are the most common reasons for needing spinal surgery [1]. In cervical radiculopathy, the predominant causes are degenerative foramen narrowing and disc herniation [1]. As vertebral discs age, they lose hydration, which weakens the annulus, leading to an increased risk of disc extrusion or herniation. Due to the posterior longitudinal ligament, the protruded disc material is typically displaced laterally, leading to direct nerve root compression. This compression exerts physical pressure and initiates a chemical inflammatory response, releasing cytokines that further irritate the nerve tissue [11].

Open discectomy, with or without spinal fusion, has been the standard approach for treating disc herniations. ACDF is still widely considered the gold standard for surgical management of cervical radiculopathy [2-6]. However, alternative treatments for cervical stenosis beyond ACDF include posterior techniques such as microscopic, endoscopic-assisted, or fully endoscopic posterior decompression and discectomy procedures [7]. Open surgical procedures often come with extended recovery periods and significant trauma due to surgery. Additionally, they carry risks such as pseudoarthrosis, loss of disc height, adjacent segment disease, and complications related to the surgical approach, including dysphagia and dysphonia [12,13]. These complications indicate the need to develop minimally invasive spine surgical procedures (MISS). MISS offer several advantages, including smaller incisions, reduced tissue disruption, minimal neural handling, less blood loss, and minimal bone removal. These benefits contribute to shorter hospital stays and quicker returns to daily activities while preserving cervical segment flexibility and stability. This stability helps alleviate postoperative axial neck pain and minimize the risk of adjacent segment disease. Additionally, endoscopic techniques can help prevent the development of epidural fibrosis, which may cause severe neurological symptoms [9].

In 1989, Tajima et al. pioneered anterior percutaneous endoscopic cervical discectomy (PECD) [14]. Depending on the disc herniation’s location, PECD can be done via an anterior or posterior approach. PECD employs a percutaneous endoscope to decompress the spinal cord or exiting nerve root, maintaining direct visibility throughout the process. This technique, which involves minimal stripping of cervical paraspinal muscles, results in significantly less postoperative neck pain. Later, newer techniques emerged, like UBE with two portals. This spinal endoscopy underwent generational changes from transforaminal and interlaminar to stenoscopy [8]. The advantages of UBE are wider viewing angles, high definition, and magnified view, more freedom of movement, reduced chance of complications, decreased need for spinal fixation, and cost-effectiveness for the patients [9]. UBE was introduced for lumbar degenerative lesions, but over time, it was used in other spinal regions like cervical and thoracic degenerative conditions. Cervical UBE is a recent advancement for treating degenerative conditions.

UBE is recommended for patients who do not respond to conservative treatments for six weeks or more. Various indications of cervical UBE are herniated cervical disc (paramedian, foraminal, or extraforaminal), foraminal stenosis, cervical radiculopathy, and degenerative cervical myelopathy [10]. Contraindications for UBE include segmental instability, cervical deformities such as scoliosis, central canal stenosis, infections, and neoplastic conditions [10]. Complications of the endoscopic procedure include injury to the dura, transient paresthesia, motor weakness, hematoma formation, incomplete decompression, and infection at the surgical site [15].

A review by Ju et al. indicates that the overall complication rate for cervical endoscopic spinal surgeries is generally low, with an estimated rate of 2% to 5% [16]. This includes various minor complications like temporary dysesthesia or minor dural tears. Open cervical spine surgeries typically have a higher complication rate, reported around 10% to 20%, depending on the type of procedure and the patient population. This higher rate is attributed to the more invasive nature of open surgeries and the associated risks [16]. Although UBE has a steep learning curve, this procedure has a lower complication rate than the available alternative open procedures.

However, in a study by Peng et al., 54 patients who underwent UBE were compared with 58 patients who had gold-standard ACDF. The study concluded no statistically significant differences in clinical outcomes or complication rates between the two procedures [17]. Further studies needed to be done with a larger sample size and longer follow-up periods to evaluate and establish the best surgical technique for cervical herniated intervertebral discs.

## Conclusions

The biportal endoscopic technique for cervical discectomy gives an excellent clinical outcome with fewer complications, minimal damage to soft tissue, and minimal blood loss. Patients also experience less postoperative neck pain because the technique causes minimal surgical trauma to the muscle ligament complex and stability of the spine, decreasing the need for spinal fixation and compromising on flexion of the neck.
